# Epidemiological Investigation of a Mortality Event in a Translocated Gopher Tortoise (*Gopherus polyphemus*) Population in Northwest Florida

**DOI:** 10.3389/fvets.2020.00120

**Published:** 2020-03-05

**Authors:** Rebecca A. Cozad, Sonia M. Hernandez, Terry M. Norton, Tracey D. Tuberville, Nicole I. Stacy, Nancy L. Stedman, Matthew J. Aresco

**Affiliations:** ^1^Warnell School of Forestry and Natural Resources, University of Georgia, Athens, GA, United States; ^2^Nokuse Plantation, Bruce, FL, United States; ^3^Southeastern Cooperative Wildlife Disease Study, College of Veterinary Medicine, University of Georgia, Athens, GA, United States; ^4^St. Catherines Island Foundation, Midway, GA, United States; ^5^Georgia Sea Turtle Center, Jekyll Island Authority, Jekyll Island, GA, United States; ^6^University of Georgia's Savannah River Ecology Laboratory, Aiken, SC, United States; ^7^Department of Comparative, Diagnostic, and Population Medicine, College of Veterinary Medicine, University of Florida, Gainesville, FL, United States; ^8^Busch Gardens, Tampa, FL, United States

**Keywords:** density, *Gopherus polyphemus*, habitat loss, reptile, starvation, stress, translocation

## Abstract

Nokuse Plantation, a 22,055 ha private conservation preserve in northwest Florida, is a recipient site for gopher tortoises translocated from development sites in Florida. Since 2006, Nokuse has received over 5,000 tortoises from multiple development sites. During 2013–2015, 52 tortoises were found sick (*n* = 14) or dead (*n* = 38) in multiple soft-release enclosures in which tortoises consistently exhibited clinical signs, with additional sick (*n* = 5) and dead (*n* = 5) tortoises presenting similarly during 2016–2017. When found alive, tortoises behaved abnormally (e.g., frequently out of burrows during cold weather, pacing along enclosure fencing), appeared emaciated, were lethargic, and had developed redness under plastron scutes. Similar numbers of male (*n* = 28) and female (*n* = 32) tortoises were recovered along with two of unidentified sex, including mainly adults (*n* = 59) and three subadults. Physical examination, blood analysis, and other diagnostics were indicative of starvation and dehydration. Most sick tortoises provided with supportive care recovered. Necropsy findings generally confirmed starvation, with no evidence of infectious pathogens or contaminants. There were no apparent differences in quality of habitat, plant community, or soil or water among affected and unaffected enclosures. Botanical surveys indicated adequate forage quality and quantity, with no poisonous exotic or native plants detected. No land management practices changed prior to this event. Analysis of epidemiological data and demographic factors from before and during this mortality event identified initial density of tortoises in the enclosures as exerting the strongest influence on detection of tortoise morbidity and mortality. We believe that the stress associated with mixing tortoises from different populations and at higher densities during translocation impacted an individual tortoise's ability to obtain or absorb adequate nutrients from foraging, ultimately leading to a wasting condition consistent with starvation. Based on our findings, we recommend a maximum of 3 gopher tortoises per ha in soft-release enclosures for translocation, but further research is warranted to investigate the complexity of stress and social pressures associated with translocation.

## Introduction

The gopher tortoise (*Gopherus polyphemus*) once occurred throughout most of the southeastern United States, particularly in areas where longleaf pine savannas were historically present (1). Direct habitat loss, habitat degradation, and historical harvesting of tortoises for their meat have caused a serious range-wide population decline, leading to federal protection of the species in the western portion of its range, and status as a candidate for federal listing in the eastern portion of the range (2–5). Over the last two decades, translocation of gopher tortoises has been increasingly used as a mitigation strategy when land development is planned, making the gopher tortoise the most widely translocated animal in the southeastern United States (6–8). Translocation, or the intentional moving of individuals from one location to another, is used in the conservation efforts for many species, although reviews of translocation programs in general have reported various levels of failure or success (6, 9–12). There have been extensive debates regarding ethical issues associated with translocation, especially for mitigation-driven translocations (13, 14).

The greatest number of gopher tortoise translocations throughout their range occur in Florida due to large-scale development of gopher tortoise habitat (15). From 1991 to 2007, Florida's mitigation policy, known as Incidental Take Permits, allowed developers to pay a mitigation fee to develop gopher tortoise habitat. This practice caused the legal entombment or killing of gopher tortoises on development sites, with estimates for number of tortoises “taken” ranging from ~84,000 to over 100,000 individuals (16–18). Although Florida Fish and Wildlife Conservation Commission (FFWCC) stopped issuing Incidental Take Permits in 2007 due to public pressure, existing permits were grandfathered at sites already slated for future development. Some gopher tortoises impacted by Incidental Take Permits are being voluntarily relocated to conservation lands and new policies since 2007 require that all gopher tortoises are translocated away from development sites. An estimated 180,000 tortoises will be translocated from new development sites in Florida by 2022 (3, 8). These mitigation-driven translocations prevent mortality of individual tortoises on the development sites by moving them to protected and managed sites with either depleted or extirpated tortoise populations (4).

Post-translocation monitoring is important for determining whether properly planned and executed translocations can successfully augment or reestablish populations of gopher tortoises. A critical component for evaluating success of translocations is the inclusion of health assessments and surveillance for pathogens (19–21). In early 2013, biologists conducting routine monitoring at one of the largest translocation sites in Florida began finding sick and dead tortoises within several soft-release enclosures. When found alive, tortoises appeared emaciated and lethargic, behaved abnormally (frequently found outside of burrows in cold weather), and had developed redness under their scutes on the plastron. Intact tortoise carcasses found fresh with no signs of predation damage also displayed the aforementioned clinical signs, suggesting that they died of disease. Other dead tortoises were also found with an intact shell, suggesting that predation was not involved. However, these carcasses were found too late to investigate the cause of death because there was little to no soft tissue remaining.

To aid future translocation and management practices at this designated recipient site, we investigated the epidemiology and potential cause(s) of the mortality event that occurred in this population of translocated gopher tortoises in northwest Florida. We analyzed gross necropsy and histopathology results from dead tortoises, diagnostic assays and the response to treatment of live tortoises, and testing for infectious pathogens. Additionally, we explored differences in soil and plant nutritional composition between two sites with different mortality rates. Finally, we used a modeling approach to determine the factors useful in predicting which individual tortoises will develop clinical signs of disease.

## Methods

### Study Site

Nokuse Plantation, a 22,055 ha private nature preserve in the Florida panhandle, is one of the largest recipient sites for translocated gopher tortoises in the state of Florida. Initial density of resident tortoises was 0.004 individuals per ha due to past habitat conversion and a long history of collection by humans for their meat before current legal protections were in place. All release sites are in sandhill communities with deep, well-drained sands [e.g., Lakeland soil; (22)] and open canopy of longleaf pine (*Pinus palustris*) and scattered turkey oak (*Quercus laevis*) with very little woody midstory. The herbaceous understory is primarily native grasses and forbs dominated by bluestem grasses (*Andropogon* spp.) and Bahia grasses (*Paspalum* spp.). Much of the sandhill was converted to farmland with varied management practices (e.g., use of center pivot irrigation and length of time that phosphate fertilizer was applied) in the late 1960s−1980s but has undergone significant restoration in the last 18 years. The abundance of herbaceous groundcover plants provide ample forage for gopher tortoises. Nokuse typically maintains a burn rotation of 3–5 years to reduce presence of a hardwood midstory, prevent succession into a mixed hardwood forest, and stimulate growth of native grasses and herbs (the main food source for tortoises). One of the sites (SR1) had not been burned for ~10 years prior to the initiation of this study, although there was not significant hardwood encroachment.

From 2006 to 2019, 5,097 gopher tortoises were translocated from development sites across Florida and released into 23 large (4–136 ha) soft-release enclosures, which has previously been shown to encourage site fidelity (23). These enclosures were constructed using a 1 m tall silt fence (temporary barrier constructed of porous fabric, typically used for sediment control) buried ~0.3 m into the ground to prevent immediate tortoise dispersion, with the silt fence removed 1–2 years after the last individual was released into the enclosure. Prior to translocation, resident tortoise density in areas where the enclosures were constructed was estimated to be 0–0.1 individuals per ha. Tortoise density inside release enclosures ranged between 0.56 and 16.36 adults per ha (Table 1). There was a roughly 1:1 ratio for adult males and females overall. Each tortoise was permanently marked with a unique notch ID [modified from Cagle (24)], and sex, donor site, and standard morphometric measurements including body weight to nearest 0.001 kg and straight carapace length (SCL) to nearest 1 mm (25) were recorded before release. Body condition indices (BCI) were calculated using the formula BCI = mass (in kg)/SCL (in mm) ^∧^3 [modified from Nagy et al. (26)]. Due to predation-related mortalities attributed to coyotes (*Canis latrans)*, 1.5 m high electric fences were installed around the perimeters of five release enclosures starting in early 2014. After the electric fences were installed, predation by coyotes was virtually eliminated without negative effects or mortality for tortoises.

**Table 1:** Release densities within soft-release enclosures and penning durations of translocated gopher tortoises at Nokuse Plantation.

**Release enclosure**	**Size (ha)**	**First tortoise released**	**Last tortoise released**	**Max penning duration (months)**	**Adults released**	**Density (adults/ha)**	**Diseased tortoises**
SN	10	5/2006	11/2006	22	29	2.87	
SS	10	5/2006	8/2006	22	36	3.56	
C1	25	10/2006	12/2007	21	57	2.27	
CB1	8	11/2006	7/2007	38	34	4.20	
CB4	134	5/2007	4/2008	34	507	3.77	
CB2	24	6/2007	10/2007	31	15	0.62	
CB5	31	6/2007	7/2007	33	183	5.87	
CB7	19	9/2007	6/2008	28	79	4.15	
C2	13	10/2007	9/2008	13	50	3.86	
CB6	89	11/2007	7/2009	29	313	3.52	
D1	7	11/2007	3/2012	13	43	6.25	
D2	4	10/2008	11/2008	7	15	3.71	
BC1	13	12/2008	9/2009	22	7	0.56	
CB9	23	10/2009	11/2011	33	251	10.88	
SR1^*^	**13**	**6/2011**	**11/2015**	**94**	**105**	**8.37**	**5**
DB1^*^	**17**	**3/2012**	**7/2013**	**40**	**278**	**16.36**	**36**
**M1**	**10**	**3/2013**	**10/2014**	**27**	**82**	**8.10**	**3**
**DB2**	**24**	**5/2013**	**6/2014**	**30**	**209**	**8.61**	**4**
**DB3**	**28**	**5/2014**	**4/2015**	**18**	**254**	**8.97**	**2**
M2	13	10/2014	7/2018	53	132	10.52	
**SR2**	**24**	**5/2015**	**3/2016**	**37**	**223**	**9.18**	**1**
WC1^**^	20	3/2016	9/2017	n/a	143	7.07	
WC2^**^	7	6/2017	5/2019	n/a	59	8.10	
M3^**^	4	10/2019	11/2019	n/a	6	1.50	
M4^**^	8	11/2019	n/a	n/a	4	0.5	

Maximum penning duration was defined as time between when the first tortoise was released into the enclosure and when the silt fence was removed. Mortality surveillance occurred in enclosures denoted in bold.

* Denotes release sites with increased monitoring efforts during 2016–2017.

** *Denotes sites with intact silt fences and/or ongoing releases at time of publication*.

### Mortality Surveillance and Investigation

**Figure 1:**
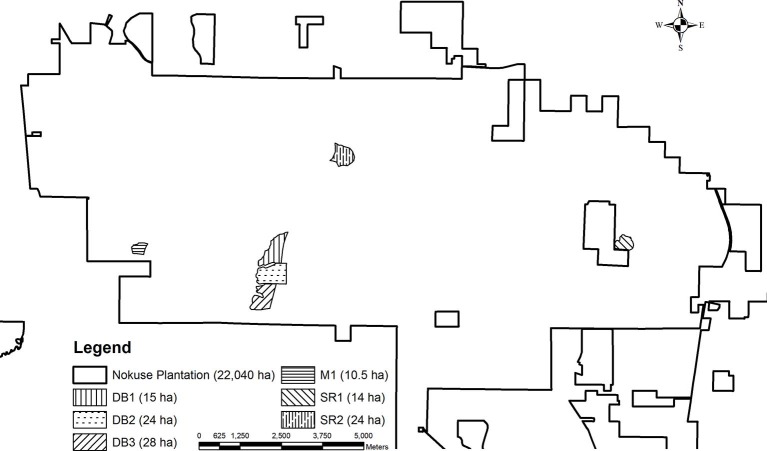
Gopher tortoise translocation release enclosures and mortality surveillance sites at Nokuse Plantation, a private conservation preserve in northwest Florida.

During 2011–2017, surveys for tortoise mortality were conducted during routine monitoring of the five enclosures with electric fences (Figure 1). Monitoring included walking the interior side of the silt fence enclosures (daily for newly constructed enclosures and weekly for others), periodic transects through interior portions of the pens (monthly), and walking the exterior of the enclosure for electric fence maintenance (weekly). Additional monitoring with high search effort in two of these enclosures (DB1 and SR1) occurred during 2016–2017, as part of a project involving trapping and radiotelemetry. When a dead tortoise was found, GPS coordinates were recorded, and the carcass or shell was recovered and brought to the field lab for individual identification (based on shell notches and morphometric measurements) and processing. Tortoises found within ~24–48 h of death based on the visual degree of decomposition were sent to a wildlife veterinarian who conducted a necropsy (TMN). If possible, live tortoises found during monitoring were captured and given a physical examination, including examination for external injuries and clinical signs of disease. If abnormalities were found, the tortoise was brought to the field office for monitoring or transported to a rehabilitation center (Georgia Sea Turtle Center [GSTC], Jekyll Island, GA) for diagnostics and treatment. Mortality and health monitoring occurred throughout the year. Analyses were performed on subsets of the total number of sick or dead tortoises encountered with clinical signs.

### Necropsy and Histopathology

A gross necropsy was completed on 21 recently dead tortoises found during mortality surveys and tortoises that died or were euthanized during rehabilitation (see section 2.6). Histopathology was only conducted on tortoises that were not autolyzed. Samples from all major tissues (skeletal muscle, heart, lung, brain, liver, kidney, spleen, gastrointestinal tract, bladder, tongue, skin, bone, and keratin) were collected for histopathological analysis. All tissues were examined microscopically by a board-certified veterinary anatomic pathologist with expertise in chelonians (NLS). Additional representative tissue samples were also collected and frozen for further testing, such as for toxicology and testing for infectious pathogens.

### Testing for Infectious Pathogens

Select tissues (liver, kidney, spleen, gastrointestinal tract, and tongue) from six dead tortoises were submitted to the University of Florida ZooMed Diagnostic Laboratory (Gainesville, FL) for screening of infectious agents common in chelonians (adenovirus, herpesvirus, and *Mycoplasma* spp.) using PCR. DNA was extracted using a DNeasy blood and tissue kit (QIAGEN Inc., Redwood City, CA) following the manufacturer's instructions and run for the agents listed above. The PCR products (DNA fragments), including a negative and previously identified positive control, were run on a 1.5% agarose gel with ethidium bromide and were visualized under ultraviolet light. Successfully amplified products were excised and purified using the QIAquick® Gel Extraction kit (QIAGEN Inc., Redwood City, CA) following the manufacturer's instructions. The purified gel pieces, including the primer, were submitted to the University of Florida Interdisciplinary Center for Biotechnology Research for Sanger sequencing. The final product was edited and compared with sequences previously entered in GenBank.

Tissues (pooled gastrointestinal tract, kidney, liver, spleen and tongue for each individual) from four tortoises were submitted to the University of Florida Wildlife and Aquatic Veterinary Disease Laboratory for ranavirus screening using quantitative PCR (qPCR).

Tissues from another 13 of the 21 necropsied tortoises were submitted to the University of Illinois Wildlife Epidemiology Laboratory (Urbana, IL), including gastrointestinal tract (*n* = 12), kidney (*n* = 11), liver (*n* = 11), spleen (*n* = 5), and tongue (*n* = 5). Tissues were screened for nine pathogens (*Anaplasma phagocytophilum, Borrelia burgdorferi, Ambystoma tigrinum* virus, Bohle iridovirus, epizootic hematopoietic necrosis virus, *Frog virus 3* [FV3-like ranavirus; FV3], *M. agassizii, M. testudineum, Salmonella enteritidis, S. typhimurium*, Testudinid herpesvirus 2, intranuclear coccidiosis) using Fluidigm qPCR (27).

Ticks were collected from tortoises during rehabilitation (*n* = 3) and during necropsy (*n* = 2). Tick samples were submitted to the University of Florida Department of Wildlife Ecology and Conservation. Conventional PCR and gel electrophoresis followed by Sanger sequencing on positive samples were used to determine the presence and identity of *Anaplasma* and *Ehrlichia* using previously published primers (28). Similarly, conventional PCR and sequencing were used to determine the presence and identity of *Rickettsia* spp. present in the tick specimens (29).

### Toxicants in Liver Tissues

Liver samples from six dead tortoises were also submitted to the Veterinary Diagnostic Laboratory (Michigan State University, East Lansing, MI) to test for presence of toxicants that were: (1) considered likely to cause the clinical signs and lesions noted; and/or (2) were potentially applied to the soils on Nokuse lands during past agricultural and silvicultural activities under previous ownership. This panel was run using inductively coupled plasma mass spectrometry and included arsenic (As), cadmium (Cd), cobalt (Co), copper (Cu), iron (Fe), lead (Pb), manganese (Mn), mercury (Hg), molybdenum (Mo), selenium (Se), thallium (Tl), and zinc (Zn). Concentrations based on wet weight are reported in parts per million (ppm). These samples were additionally evaluated by gas chromatography mass spectrometry and screened against the Wiley 9 Mass Spectrometry library, which includes >500,000 common agricultural pesticides and herbicides, as well as persistent organic pollutants.

### Diagnostics and Rehabilitation of Live Tortoises

Nineteen tortoises found alive but sick (displaying clinical signs such as emaciation, lethargy, abnormal behavior, and/or redness under the plastron scutes) were transported to the GSTC for diagnostics and treatment. Standard morphometrics were recorded at admission. Each tortoise received a complete health assessment by a zoological medicine board-certified specialist (TMN). Body condition indices were calculated for comparison with data from initial release. Ectoparasites were documented and, if present, collected, and stored in alcohol.

Using a sodium heparinized syringe, blood was collected from either the jugular or brachial vein for a suite of blood analyses to assess overall health of the individual tortoise. The volume of blood collected did not exceed 0.5% of the tortoise's body weight. Whole blood was transferred to a microhematocrit tube and centrifuged (Hemata-Stat II, Separation Technology Inc., EKF Diagnostics) to measure packed cell volume (PCV) and plasma color, and a refractometer was used to measure plasma total solids (TS). Within 30 min of collection, a small amount of heparinized whole blood was used to make blood films for review, including white blood cell (WBC) estimate and differential, and morphological evaluation, performed by a board-certified veterinary clinical pathologist (NIS). Remaining blood was centrifuged (Triac centrifuge, Primary Care Diagnostics, Becton Dickinson Company) to harvest plasma. A biochemical panel was performed on all plasma samples with an AU480 chemical analyzer (Beckman Coulter Inc., Brea, CA) by IDEXX Laboratories (Westbrook, ME): aspartate aminotransferase (AST), creatine kinase (CK), albumin, total protein, globulin, blood urea nitrogen (BUN), cholesterol, glucose, calcium, phosphorus, chloride, potassium, sodium, uric acid, and triglycerides.

During rehabilitation, tortoises were kept in housing maintained between 23 and 32°C (30). All tortoises received antimicrobials [enrofloxacin (Baytril, Bayer HealthCare LLC., Shawnee Mission, KS)] and fluid therapy [Lactated Ringer's Solution (Abbott Laboratories Inc., Chicago, IL)]. Dependent on initial diagnostics, tortoises also received supplementation with B vitamins and/or iron. Tortoises suspected to have gastric ulcers were treated with sucralfate (TEVA Pharmaceuticals USA Inc., North Water, PA), and tortoises with ocular discharge or palpebral edema were treated with tetracycline ophthalmic ointment (Terramycin, Zoetis Inc., Kalamazoo, MI). All tortoises were closely monitored for eating and defecation and were frequently soaked in shallow water to promote hydration. Tortoises that did not eat on their own were provided nutritional support through a red rubber feeding tube with a reptile herbivore complete diet (Emeraid IC Herbivore, Emeraid LLC., Cornell, IL).

### Forage and soil sampling and testing

Forage and soil samples were collected at approximately 30 active gopher tortoise burrows in each of two enclosures, DB1 and SR1 (see Figure 1), during July and August 2017. DB1 was a site of high disease-related mortality, while SR1 had no documented disease-related mortality and was chosen as a site for comparison. At each selected burrow, a 1-m^2^ quadrat was placed ~5 m away at a randomly selected angle (0–180°) from the mouth of the burrow. If the randomized angle resulted in placement around the base of a tree or shrub, the next angle was chosen.

All above-ground herbaceous vegetation within the quadrat was cut at the soil line, collected, and transported to the lab for processing. Vegetation samples from each enclosure were separated into two types: (1) grasses and grass-like plants and (2) non-grass mixed forbs and legumes. For each enclosure, samples of each vegetation type collected from individual burrows were thoroughly mixed by group to create a composite mixture (e.g., DB1 mixed grasses, DB1 mixed forbs and legumes, etc.). Two composite samples from each forage group from each site were submitted for analysis through wet chemistry procedures (Dairy One Forage Laboratory, Ithaca, NY, see Appendix A in Supplementary Material for specific procedure details).

Soil cores of 30 cm were collected from the center of each quadrat using a soil auger. Each soil core sample was then separated into a top (depth = 0–15 cm) and bottom (depth = 16–30 cm) layer. Samples were then pooled by enclosure and depth layer and thoroughly mixed to create a composite sample (*n* = 4). Total elemental analysis of soils was performed for each composite sample by the University of Georgia Soils Lab (Athens, GA), where levels of the following elements were measured through acid digestion: phosphorus (P), potassium (K), calcium (Ca), Fe, magnesium (Mg), Mn, Mo, boron (B), Cu, nickel (Ni), sodium (Na), sulfur (S), Zn, aluminum (Al), As, Cd, chromium (Cr), and Pb. Element concentrations based on dry weight are reported in ppm.

### Statistics and Modeling for Predictor Variables for Disease

Sex of diseased tortoises was compared using a Welch two sample *t*-test in statistical program R [version 3.4.2; (31)]. All blood data for rehabilitated tortoises were compared with data from free-ranging (translocated) healthy gopher tortoises from Nokuse Plantation using a Welch two sample *t*-test in R. Forage data were compared using Analysis of Variance (ANOVA) in *R* to test for effects of site, forage type, and a site^*^type interaction. Soil data were compared between sites (DB1, SR1) using a Welch two sample *t*-test in *R*.

To identify potential variables useful for predicting morbidity and mortality, all data collected for tortoises translocated to five of the release enclosures (including enclosures with known disease-related mortalities) was compiled. Individual tortoises encountered either dead or alive during mortality surveillance were designated as either “diseased” or “healthy.” Diseased animals were those tortoises that experienced disease-related mortality or underwent rehabilitation. Individuals detected after January 2016 were excluded from this modeling analysis, as they were encountered during non-routine monitoring with high search effort (e.g., trapping and radiotracking). Data for the models included donor site location and characteristics, recipient site (release enclosure) characteristics, and individual tortoise data. Donor site variables (when available) included donor site, donor county, number of tortoises in cohort (in this study defined as tortoises collected from the same donor site and moved at the same time), donor site latitude, change in latitude from donor site to recipient site, presence of a “sick” cohort member (binary response), and number of sick tortoises in the cohort. Recipient site variables included initial stocking density (tortoises/ha), number of distinct donor populations in enclosure, and days since last burn. Individual tortoise variables included sex, SCL, initial BCI at time of release (calculated using SCL and mass), days since release (into soft-release enclosure; includes time after silt fence was removed), month encountered, and season encountered (assigned using month encountered; spring = March, April, May; summer = June, July, August; fall = September, October, November; winter = December, January, February).

Biologically relevant combinations of variables within each candidate set of models were estimated with logistic regression, and model fit was assessed using Akaike Information Criterion [AIC; (32)] as a ranking variable in R. These were run in a fixed-effects model with a binomial distribution to determine relationship between the variables and the binomial response outcome of detection (1) or absence (0) of disease.

Three separate sets of candidate models were evaluated initially to determine whether any of these sets of characteristics were important for predicting the detection of disease (Supplementary Table). Each set of candidate models was focused on a set of characteristics—e.g., donor site, recipient site, or host (tortoise). Highly correlated variables (such as static categorical variables pertaining to site) were excluded from use in the same model. Results from these models were also compared with a null model (intercept only). A global model was not included, as there were correlated variables that could not be run in the same model.

Variables included in the top model(s) for each candidate set were then compiled to create a final set of combination models. Models that were tested in this final set included only variables that made biological sense when combined. Model results were evaluated using AIC for top model selection.

## Results

Between April 2013 and April 2017, 62 tortoises were found sick or dead with the cause attributed to disease. We defined the morbidity and mortality event as the period between April 2013 and November 2015, during which most diseased tortoises (*n* = 52; 84%) were encountered, although there were additional diseased tortoises (*n* = 10; 16%) found between January 2016 and April 2017 that are also included in some analyses. Tortoises were found sick or dead in all months except February, and almost all (59/62) were adults. There was no difference (*p* = 0.2) in number of males (*n* = 28) and females (*n* = 32) found sick or dead, and mean SCL was 271 ± 35 mm SD. The timing between a tortoise's release and when it was found sick or dead varied between 0 and 5 years. Two tortoises were assessed and determined to be sick upon arrival and was transported for rehabilitation instead of being released; all others were found sick or dead years after their initial release. Mortalities were initially confined to one site (DB1; 15 ha; April to December 2013) but several tortoises were later found sick or dead in an adjacent site, DB2 (24 ha; December 2013). Sporadic mortalities also occurred in other release sites, sometimes several miles away from DB1 and DB2. Most tortoises during the event were found in DB1 (36/52; 69%), with the others found in DB2 (*n* = 4), DB3 (*n* = 2), and M1 (*n* = 3). Other tortoises were from unidentified enclosures (*n* = 3), other sites on Nokuse (*n* = 2), or were never released (*n* = 2).

### Gross Necropsies and Histopathology

Of the 21 animals that were submitted for necropsy, histopathology, and ancillary testing, we observed signs consistent with inanition or malnutrition [e.g., poor body condition (*n* = 17), hepatic (*n* = 4) and/or skeletal muscle (*n* = 14) atrophy, atrophy of adipose tissue within the bone marrow (*n* = 1)], mild to severe osteopenia of the plastron (*n* = 2), hemosiderosis (accumulation of iron) in the liver (*n* = 13), or no gross or microscopic evidence of infectious disease (*n* = 21). Emaciation, muscle atrophy, empty (or nearly empty) gastrointestinal tract, and liver hemosiderosis were the most common findings for dead tortoises.

The esophagus, stomach and small intestine of many tortoises that died were devoid of forage contents: seven had completely empty stomachs, five had distended stomachs filled with gas or clear fluid, and an additional two had abnormal material and mucous present in the stomach. Two tortoises had only tube-feeding formula in the intestinal tract (from supportive care), and only one necropsied tortoise had grass present in the stomach. One tortoise also had necrotic mucosa and another had necrotic-like material present in the gastrointestinal tract. Some of the tortoises had fecal material in the colon and gas in the gastrointestinal tract which was attributed to gastrointestinal stasis or ileus, a common finding in sick chelonians. None of the gastrointestinal tracts contained unusual plants or foreign objects. An enlarged or distended urinary bladder with amber colored urine and gray urates was also documented in 10 (48%) of the necropsied tortoises, which is consistent with dehydration and/or starvation in tortoises (33).

### Testing for Infectious Pathogens

The only pathogen detected in samples submitted to the University of Florida was *Mycoplasma* spp., where DNA was detected in three of six tortoises. Histopathology of the upper respiratory tract of two tortoises was consistent with mild to severe upper respiratory tract disease (URTD).

Low levels of FV3 DNA (mean = 0.1433 copies/ng DNA, SD ± 0.085) were detected in the gastrointestinal tract of three tortoises, while other samples submitted to the University of Illinois were negative for all other pathogens tested (34). No other pathogens were detected in tissue samples.

Ticks were identified as gopher tortoise ticks (*Amblyomma tuberculatum*). No tortoise pathogens were detected in any of the submitted tick samples.

### Liver Toxicants

Liver concentrations for As, Cd, Co, Cu, Fe, Pb, Mn, Hg, Mo, Se, and Tl were within the expected range based on the limited reference ranges for liver mineral values in tortoises for the Michigan State University Veterinary Diagnostic Laboratory (Table 2; Buchweitz, pers. comm.). No toxic organic compounds screened for in the Wiley 9 MS library were detected in any of the liver samples.

**Table 2:** Liver toxicant concentrations in parts per million (ppm) for selected elements (arsenic [As], cadmium [Cd], cobalt [Co], copper [Cu], iron [Fe], lead [Pb], manganese [Mn], mercury [Hg], molybdenum [Mo], selenium [Se], thallium [Tl], and zinc [Zn]) in liver tissue of six translocated gopher tortoises (*Gopherus polyphemus*) that died during a mortality event from 2013 to 2015 at Nokuse Plantation, a private preserve in northwest Florida.

		**Selected elements (ppm)**											
**ID**	**SEX**	**As**	**Cd**	**Co**	**Cu**	**Fe**	**Pb**	**Mn**	**Hg**	**Mo**	**Se**	**Tl**	**Zn**
2021	F	<0.03	0.12	<0.03	2	1,397	0.03	2.64	<0.13	0.8	0.09	<0.03	39
2172	M	<0.03	0.19	<0.03	4.4	1,577	<0.03	2.11	<0.13	1.12	0.72	<0.03	93
2197	F	0.05	0.22	<0.03	2.1	2,772	0.09	3.7	0.06	1.03	0.18	<0.03	42
2274	F	<0.03	0.93	<0.03	3	1,271	<0.03	7.64	<0.13	0.62	0.19	<0.03	88
2552	M	<0.03	0.37	<0.03	4.4	1,133	<0.03	1.34	<0.13	0.7	0.18	<0.03	20
307	F	<0.03	0.93	<0.03	3	1,271	<0.03	7.64	<0.13	0.62	0.19	<0.03	88

*Concentrations based on wet weight values*.

### Tortoises in Rehabilitation

**Figure 2:**
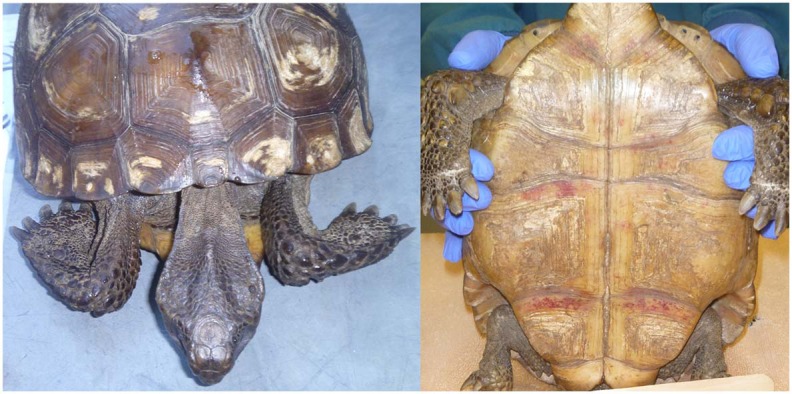
Clinical presentation documented in gopher tortoises during rehabilitation at the Georgia Sea Turtle Center included emaciation (**Left**) and hemorrhaging under the scutes on the plastron (**Right**). Photos provided by T. Norton.

Nineteen tortoises were found sick but still alive between August 2014 and April 2017 and admitted to the GSTC for medical care. Physical examinations of all tortoises indicated poor body condition, lethargy, and severe weakness. Some also had obstructed nares, nasal discharge, pale mucous membranes, and varying degrees of red discoloration under the scutes of the plastron suggestive of hemorrhage (Figure 2); no other consistent clinical abnormalities were found. The neurological function of tortoises was assessed frequently and considered normal. Body condition indices and CBCs were obtained on 11/19 tortoises and plasma biochemistry testing was performed on 11/19 tortoises within 14 days of arrival at the GSTC and compared with previously published blood analyte data from other studies (Table 3). One tortoise had blood drawn on two sample dates (day 0 and day 14). Blood sampling was delayed to avoid further stressing of severely compromised tortoises. Compared to clinically normal free-ranging tortoises from Nokuse Plantation, tortoises admitted for rehabilitation had, on average, significantly lower body weight (*p* < 0.01), BCI (*p* < 0.01), PCV (*p* = 0.02), immature heterophils (*p* < 0.01), lymphocytes (*p* < 0.01), total solids (*p* < 0.01), total protein (*p* < 0.001), albumin (*p* < 0.01), globulin (*p* < 0.01), cholesterol (*p* = 0.03), glucose (*p* < 0.01), calcium (*p* = 0.02), and potassium (*p* = 0.02), and significantly higher AST (*p* = 0.04) and BUN (*p* < 0.01) (Table 4). The anemia in these tortoises was moderate to severe and non-regenerative. No hemoparasites or other infectious agents were identified in any of the blood films and plasma color, when noted, was clear. Seven of 12 tortoises had mild to moderate heterophil left-shifting.

**Table 3:** Comparative hematology and blood chemistry data from gopher tortoises (*Gopherus polyphemus*) translocated to Nokuse Plantation sampled during rehabilitation within 14 days after admission in comparison to a free-ranging population translocated from Georgia to South Carolina (35) and a free-ranging population in Georgia (36).

**Hematology**	**Rehab (<14 days)(*****n*** **=** **12)**			**Hernandez et al**. **(****35****)** **(*****n*** **=** **10)**				**Taylor and Jacobson** **(****36****)** **(*****n*** **=** **13)**		
	**Mean**	**Min**	**Max**	**Mean**	**Min**	**Max**		**Mean**	**Min**	**Max**
Packed Cell Volume	15	8	25	25	14	29		23	15	30
White blood cell concentration (× 10^3^/ul)	12.79	3.80	32.00	10.28	4.20	17.6		15.70	10.00	22.00
Heterophils (× 10^3^/ul)	6.75	1.50	21.00	4.81	1.60	10.9		^**^4.76	1.57	8.95
Immature Heterophils (× 10^3^/ul)	0.31	0	1.70							
Lymphocytes (× 10^3^/ul)	2.71	0.34	4.60	1.43	0.29	5.46		8.89	5.02	12.4
Monocytes (× 10^3^/ul)	1.60	0.13	3.70	0.19	0	0.72		1.16	0.47	20.88
Eosinophils (× 10^3^/ul)	0.81	0	2.60	0.56	0	1.51		^**^ (see heterophils)		
Basophils (× 10^3^/ul)	0.77	0	1.90	3.37	1.25	7.92		0.90	0.31	1.73
Heterophil:Lymphocyte Ratio	2.70	0.71	8.08	5.23	1.61	10.69				
**Blood chemistry**	**Rehab (<14 days) (*****n*** **=** **11)**			**Hernandez et al**. **(****35****)** **(*****n*** **=** **14)**				**Taylor and Jacobson** **(****36****)** **(*****n*** **=** **variable)**		
	**Mean**	**min**	**max**	**Mean**	**min**	**max**	**n**	**Mean**	**min**	**max**
Total Protein (g/dL)	2.6	1.5	3.9	4.5	3.5	5.8	17	3.1	1.3	4.6
Albumin (g/dL)	0.4	0.1	0.9	1.2	1.0	1.5	17	1.5	0.5	2.6
Globulins (g/dL)	2.2	0.9	3.3							
AST (U/L)	240	53	650	163	87	252	17	136	57	392
Creatine Kinase (U/L)	3,117	31	27,231	1,408	55	5,705	13	160	32	628
Blood Urea Nitrogen (mg/dL)	89	15	229					30	1	130
Cholesterol (mg/dL)	36	9	70	121	10	282	13	76	19	150
Glucose (mg/dL)	72	8	134	131	81	197	16	75	55	128
Calcium [Ca] (mg/dL)	9.4	8.2	11.7	15.7	11.4	23.2	10	12	10	14
Phosphorus [P] (mg/dL)	2.0	1.0	4.1	2.8	1.7	6.1	17	2.1	1.0	3.1
Ca/P ratio	5.4	2.2	8.4							
Chloride (mEq/L)	93	24	121	92	88	98	17	102	35	128
Potassium (mEq/L)	4.0	0.9	6.8	4.0	3.3	5.3	17	5.0	2.9	7.0
Sodium (mEq/L)	119	30	151	126	121	130	17	138	127	148
Uric Acid (mg/dL)	7.2	2.0	26.7	2.6	1.4	4.8	17	3.5	0.9	8.5
Triglycerides (mg/dL)	14	2	33	177	29	523	12	32	0	178

** *Heterophils and eosinophils combined as reported in reference*.

**Table 4:** Differences in body condition, plasma biochemistry, and hematology data between clinically abnormal translocated gopher tortoises (*Gopherus polyphemus*) in rehabilitation and clinically normal free-ranging tortoises at Nokuse Plantation.

	**Rehab (*****n*** **=** **12)**			**Free-Ranging (*****n*** **=** **83)**			
**Parameter**	**Mean**	**Min**	**Max**	**Mean**	**Min**	**Max**	***p***
Weight (kg)	2.77	1.90	4.00	3.76	1.56	5.73	<*0.01*
Carapace length (cm)	25.97	23.10	30.80	27.70	21.00	31.70	*0.04*
Body condition index	0.16	0.14	0.19	0.18	0.14	0.22	<*0.01*
Packed cell volume (%)	15	8	25	26	12	43	*0.02*
White blood cell concentration (× 10^3^/ul)	12.79	3.80	32.00	16.25	7.20	35.60	*0.2*
Heterophils (× 10^3^/ul)	6.75	1.50	21.00	7.80	1.70	21.00	0.56
Immature heterophils (× 10^3^/ul)	0.31	0	1.70	0.65	0	3.50	<*0.01*
Lymphocytes (× 10^3^/ul)	2.71	0.34	4.60	4.27	1.60	9.40	<*0.01*
Monocytes (× 10^3^/ul)	1.60	0.13	3.70	1.40	0	4.30	*0.64*
Eosinophils (× 10^3^/ul)	0.81	0	2.60	1.48	0	23.00	*0.11*
Basophils (× 10^3^/ul)	0.77	0	1.90	0.96	0	3.50	*0.41*
Heterophil:Lymphocyte ratio	2.70	0.71	8.08	1.98	0.38	5.24	*0.30*
	**Rehab (n=11)**			**Free-Ranging (n=88)**			
Total Solids (g/dL)	2.6	1.0	4.0	3.6	2.1	5.6	<*0.01*
Total Protein (g/dL)	2.6	1.5	3.9	3.7	1.3	6.4	<*0.01*
Albumin (g/dL)	0.4	0.1	0.9	0.8	0.1	1.9	<*0.01*
Globulin (g/dL)	2.2	0.9	3.3	3.0	1.2	5	<*0.01*
AST (U/L)	240	53	650	96	11	241	*0.04*
CK (U/L)	3,117	31	27,231	1,357	33	8,341	*0.49*
BUN (mg/dL)	89	15	229	4	0	70	<*0.01*
Cholesterol (mg/dL)	36	9	70	54	6	139	*0.03*
Glucose (mg/dL)	72	8	134	117	22	264	<*0.01*
Calcium [Ca] (mg/dL)	9.4	8.2	11.7	10.6	0	18.1	*0.02*
Phosphorus [P] (mg/dL)	2.0	1.0	4.1	2.6	0.7	9.1	*0.06*
Ca/P ratio	5.4	2.2	8.4	4.5	0	10.9	*0.22*
Chloride (mEq/L)	93	24	121	103	35	270	*0.26*
Potassium (mEq/L)	4.0	0.9	6.8	5.0	2.0	9.9	*0.02*
Sodium (mEq/L)	119	30	151	135	49	276	*0.15*
Uric Acid (mg/dL)	7.2	2.0	26.7	3.4	0.6	9.9	*0.12*
Triglycerides (mg/dL)	14	2	33	21	2	252	*0.19*

*AST, aspartate aminotransferase; CK, creatine kinase; BUN, blood urea nitrogen*.

When tortoises were treated with supportive care (fluid therapy, warmth, vitamins, and other supplements), nutritional support, and antibiotics, some improved and showed increasing appetite and normal food consumption. Seven animals (37%) died or were ultimately euthanized because of their deteriorating condition, while the other 12 (63%) recovered at the GSTC. Once recovered, tortoises were returned to Nokuse Plantation, where they were released into a separate, smaller enclosure and provided with supplemental food. After 11–15 months, seven of the 12 recovered tortoises (58%) were successfully recaptured, reassessed, and released in a new larger enclosure. Two of the tortoises that were rehabilitated and released were later found dead in or near their burrows, but the cause of their death remains undetermined.

### Nutrients in Forage Plants and Minerals in Soils

Only significant differences in the plant nutritional composition between the two study sites (DB1, SR1) are reported here. Starch (*p* < 0.03), Ethanol Soluble Carbohydrates (ESC, simple sugars; *p* = 0.007), crude fat (*p* < 0.03), potassium (*p* < 0.001), manganese (*p* = 0.03), and Dietary Cation-Anion Difference (DCAD; *p* = 0.001) were all higher in the DB1 site. Phosphorus (*p* < 0.02), magnesium (*p* < 0.02), sulfur (*p* = 0.007), and chloride ion (*p* < 0.001) values were higher in the SR1 site. There were no significant differences between sites for any of the soil minerals analyzed.

### Models Exploring Predictors of Disease

The top model for the recipient site candidate set included initial density and days since burn as variables, with a model weight of 0.972. Within the candidate set for donor site, two models captured most of the weight, with the number of diseased tortoises in cohort included as a variable in both models. That variable alone accounted for the majority of the weight (0.726), followed by the secondary model which also included donor region (model weight = 0.257). The top model in the host candidate set included initial BCI, season encountered, and days since release (model weight = 0.459) followed by just season encountered as a variable (model weight = 0.278).

Three models with the combined variable types captured almost all of the weight of the models, with the two variables of initial density and season encountered included in all three (Table 5). The variables of initial BCI, days since release, and days since burn were also included in one or more of the models that captured most of the weight. Sick tortoises were detected more frequently in enclosures with higher initial densities (8.1–16.36 adults/ha) than those with lower initial densities (0.56–8.1 adults/ha). Disease was detected most frequently in the fall and winter, indicating that there is likely a seasonal component in presence of disease or its detection. There was a bimodal effect from days since last burn, where tortoises were also more likely to be detected either 1–3 years before a prescribed burn, or within the first 2 years following a burn. Tortoises were more likely to be detected with disease within the first 3 years after release. Tortoises detected with disease had an initial BCI of 0.15–0.20, which was in the intermediate range of individuals represented in the data.

**Table 5:** Akaike Information Criterion (AIC) table for top logistic regression models demonstrating variables for predicting detection of disease in translocated gopher tortoises (*Gopherus polyphemus*) at Nokuse Plantation, a private conservation preserve in northwest Florida.

**Model**	**K**	**AIC**	**ΔAIC**	**Model Wt**
initial_bci+days_since_release+initial_density+season	8	153.5	0	0.405
days_since_release+initial_density+season+days_since_burn	8	153.8	0.2	0.36
initial_bci+days_since_release+initial_density+season+days_since_burn	9	155.1	1.6	0.184
null model (intercept only)	1	368.8	215.3	0

*BCI, body condition index*.

## Discussion

This study documents the investigation of a mortality event of gopher tortoises exhibiting evidence of starvation and dehydration presumptively resulting from higher tortoise density and associated chronic stress after translocation. Previous studies have demonstrated that translocated gopher tortoise populations with appropriate management can have very high rates of retention and nearly 98% annual apparent survival more than 10 years post-release (7, 37). Current approaches for gopher tortoise translocation rely on previous studies to improve short-term translocation success, such as soft-release enclosures to increase site fidelity (23, 38). While the mortality event was pronounced enough to warrant an investigation, the apparent annual survival of adults for the affected enclosures (and Nokuse as a whole) is 0.89–0.98 (Aresco, unpubl. data), which is on par with or greater than apparent annual survival rates of 0.87–0.99 reported for adult wild (39–41) and translocated (7, 37, 42) gopher tortoise populations. The time from introducing a tortoise to Nokuse Plantation to the tortoise becoming ill or being found dead did not seem to affect likelihood of mortality during this event, as tortoises both recently introduced and those that had been there for years died. There was also no significant difference between number of diseased males and females, suggesting that detection of disease was not biased by sex. The majority of tortoises were also found during the cooler months, when they would be expected to be less active. However, aberrant thermoregulatory behavior may have been easier to detect during cooler months, as tortoises with poor health may spend significantly more time basking or being out of their burrows at inappropriate temperatures (43–45) rather than undergoing winter dormancy (46). This behavior of emergence or basking in cooler weather may suggest that the tortoises found in the fall and winter were potentially attempting to behaviorally induce a fever, which gopher tortoises have been shown to do when their immune system is challenged (47).

There were several commonalities among tortoise necropsy results. Tortoises were emaciated, had moderate to severe muscle atrophy, a mostly empty gastrointestinal tract, and hemosiderin accumulation in the liver. The combination of these factors suggests that these animals had not been eating for an extended time prior to death, although the cause remains unknown. When found alive, individuals exhibited muscle atrophy, had poor body condition, sub-keratin hemorrhage of the plastron, and bloodwork findings consistent with a catabolic state and consequent starvation. Although starvation due to lack of adequate forage within the release sites seems unlikely given the abundance and diversity of suitable forage plants present in all of the known affected sites and the fact that many other tortoises were unaffected, underlying disease and/or chronic stress could lead to decreased foraging and consequent malnutrition (48, 49).

There are no other published studies that include data for minerals in livers for gopher tortoises; thus, comparisons were drawn from the closely related desert tortoise (*Gopherus agassizii*), which occurs in southwestern US deserts. Hepatic iron concentrations from necropsied gopher tortoises were similar to those in desert tortoises with clinical signs of URTD (p=0.99) but significantly higher than a control group of healthy desert tortoises (*p* = 0.01) (50). Increased hepatic iron concentrations may be attributed to iron accumulation from red blood cell breakdown and inability to reabsorb the iron from hemoglobin (50). This is consistent with our necropsy findings of hepatic hemosiderosis and could result from cachexia, or weakness and muscle wasting due to severe chronic illness (51). A mortality investigation for desert tortoises in the Mojave Desert following periods of drought found similar necropsy and histopathology results, including hepatic hemosiderosis (52). Based on the toxicological assays of liver tissues, ingestion of toxicants due to past agricultural and silviculture practices seems unlikely as the cause of disease and mortality in these tortoises.

Other than *Mycoplasma* spp., the causative agent linked to URTD, there is scant information on the diseases and pathogens of wild gopher tortoises. However, none of the histopathological findings or infectious pathogen assays provide evidence to suspect an infectious pathogen as the cause of these mortalities. We did detect *Mycoplasma* spp. DNA in tissue from three tortoises and FV3 DNA at low levels in the gastrointestinal tracts of three tortoises. However, the severity and the inconsistent frequency of lesions noted in necropsied tortoises do not indicate that mortalities were caused by URTD or by *Mycoplasma* spp. infection. Additionally, as ranavirus was not detected in other organs, the low number of copies present in the three positive samples, and most importantly, the lack of a demonstrable host response, it is also unlikely that ranavirus was the cause of mortality. The three tortoises instead may have been asymptomatic carriers of ranavirus, or the virus was simply passing through the gastrointestinal tract (34). Novel pathogens remain a possibility and continued testing to eliminate infectious agents as the cause of future mortalities is recommended.

The data on body condition, physical examination, plasma biochemistry, and hematology of gopher tortoises evaluated during rehabilitation are consistent with chronic malnutrition. Blood chemistry data revealed that total protein, total solids, albumin, globulins, cholesterol, glucose, calcium, and potassium were significantly lower when compared with free-ranging clinically normal tortoises of the same study population, and AST and BUN were higher. Similar trends were observed for these analytes when comparing diseased tortoises from this study with previously reported blood analytes data for free-ranging tortoises elsewhere, with the additional observation of comparatively higher CK and uric acid in our study tortoises (35, 36). The following interpretations are based on general principles of blood data interpretation in reptiles (53). Anemia in reptiles can be the sequela of various underlying causes, such as chronic disease and/or inflammatory disease (e.g., from infectious or non-infectious causes), but also has been associated with starvation and dehydration in desert tortoises (52). Considerations for generalized low plasma proteins (i.e., panhypoproteinemia which is defined as low concentrations of total protein, albumin, and globulins) in context of other abnormalities in blood variables include chronic malnutrition resulting from decreased protein synthesis and/or increased catabolism, in addition to malabsorption and/or maldigestion, and less likely hepatic insufficiency or protein loss from renal disease. Hypocalcemia and hypokalemia are often also associated with anorexia or malnutrition in reptiles. Cholesterol was lower in sick tortoises admitted for treatment when compared to free-ranging tortoises, presumptively due to anorexia, malnutrition and/or malabsorption. Increased BUN may be the result of increased protein catabolism or intestinal hemorrhage. Although both AST and CK were mildly elevated in tortoises during rehabilitation, only AST was significantly higher, possibly due to the longer half-life of AST, suggesting muscle wasting. Hyperuricemia (>3 mg/dL uric acid) was observed in 10 of 12 tortoises, indicating dehydration. Given the evidence of dehydration, the observed panhypoproteinemia was likely more severe than actually reflected in plasma protein concentrations. Hematological data of rehabilitating tortoises was consistent with anemia of chronic and/or inflammatory disease. Clinically normal free-ranging Nokuse tortoises had higher immature heterophils and lymphocytes than rehabilitating tortoises, suggesting active immune stimulation. The presence of comparatively higher immature heterophils, monocytes, and eosinophils and wide range of leukocytes indicating leukopenia or leukocytosis in rehabilitating tortoises compared to free-ranging tortoises from other studies suggest possible immunosuppression from bone marrow depression to active systemic inflammation, respectively, presumptively reflecting various stages of being affected by stress, non-specific inflammation, and/or immunosuppression. Given the context of necropsy and histopathological findings of tortoises that died in which there was no evidence of infectious disease, the clinicopathological abnormalities and clinical findings in rehabilitating tortoises are consistent with chronic malnutrition, starvation, and dehydration. Together these results support that these tortoises were not eating sufficient amounts, were eating nutritionally deficient forage, or were unable to properly absorb nutrients from the forage.

There are no apparent differences in the quality of habitat, gopher tortoise forage, or soil type and composition among release sites (Aresco, pers. comm.). Examination of the affected enclosures (i.e., those experiencing mortalities) by a botanist did not detect any poisonous exotic or native plants (A. Schotz, AL Natural Heritage Program, unpubl. report) and there is an ongoing investigation on the plant community composition. Forage nutritional assays showed that the forage tested from DB1 (the site with the highest mortality) had higher amounts of non-fibrous carbohydrates such as starches and sugars, which suggests more readily available energy was present in plants from that site when compared to those from the site where no mortalities occurred (SR1). No land management practices had recently changed prior to this mortality event. Although the specific historical farm practices differed among sites, no discernible correlation between farming practice and tortoise mortality has yet to emerge (Aresco, pers. comm.), and soil and liver samples tested did not contain elevated levels of agriculture-related toxicants. The only habitat management related factor that emerged as important for detection of disease was the time since last prescribed burn. This variable had a bimodal effect on probability of detection of disease. Tortoises were likely to be detected with disease within the year following a prescribed burn, although this is likely due to their increased visibility following the removal of the understory vegetation, similar to the increased detection of tortoise burrows post-burn (54). Additionally, diseased tortoises were also more likely to be detected within 1 year prior to the last prescribed burn, which is functionally 3–5 years after a burn based on the current burn rotation. This suggests that burn frequency should at the very least be maintained at 3–5 years, although it may be beneficial to burn more frequently. Fire or other frequent disturbance is required within this ecosystem to maintain the open canopy and sparse midstory that gopher tortoises prefer, and lack of frequent fire on the landscape can lead to suboptimal habitat and foraging conditions (41, 55).

Our highest ranked models suggest that certain recipient site, donor site and individual tortoise characteristics were important in detecting diseased tortoises. Season was an important component of several of the top models, with diseased tortoises more likely to be detected in fall and winter than in other seasons. As previously discussed, diseased tortoises may have been more detectable when abnormal thermal behavior is more apparent. Although no biological cause for a seasonal pattern in actual disease status could be determined, seasonal differences in immunity have been reported in ectotherms, which may be particularly susceptible during times of thermal instability such as onset of dormancy (56, 57). In gopher tortoises, the number of circulating lymphocytes and bactericidal ability are significantly reduced in fall and winter compared to other seasons (58).

Initial tortoise density in release pens was the most important variable for detecting disease. The models indicated that diseased tortoises were found most frequently in enclosures with higher densities. Although we did not detect a pathogen responsible for the mortalities, holding animals at higher densities increases the probability of pathogen transmission and disease, which can be exacerbated in highly social species with complex social structures, such as the gopher tortoise (59, 60). Gopher tortoise movement and behavior patterns are affected by density, and translocated individuals also tend to have increased movements and home ranges (23, 61, 62). The increased movement exhibited by translocated tortoises also leads to increased contact opportunities for transmission of potential pathogens (21). Given the complex social structure of gopher tortoise populations, it is logical that density may influence the stress (and health) exhibited by individuals after translocation (21). Overcrowding was also believed to be a contributing factor in mortalities of desert tortoises exhibiting similar clinical signs as in this study (52).

Other variables included in the top models included days since release and initial BCI. Tortoises were more likely to be detected with disease within the first 3–4 years after release, with the probability of detection of disease decreasing after that time point. The initial time post-translocation while a tortoise is acclimating to a novel environment (“establishment phase”) is also a time of high stress, and therefore higher health risk (49, 63). Our models suggest that once a tortoise survives more than 3–4 years post-translocation, the likelihood that they will be found diseased is much lower.

Condition indices have been used to quantify health and estimate energy reserves in various taxa, but may not be the most reliable assessment of body condition, especially in chelonians (64, 65). The method used to calculate BCI in this study (using only SCL and mass) does not accurately represent the approximate volume of the tortoise. Additionally, BCIs can be affected by several factors, including hydration and reproductive status (26, 64). The variable size and shape of individual tortoises also suggests that weight alone is not a good indicator of health and condition, such that a method that evaluates muscle mass and fat deposits may be a better option (66). Regardless, individuals with a low initial body condition (and therefore energy reserves) may be more likely to remain at a lower body condition, especially under stressful conditions. Tortoises with lower energy reserves may also be at a higher risk for deteriorating health, particularly when evaluated in conjunction with the other highlighted variables.

Our results may serve as a cautionary tale for future translocation efforts, as new pathogens and diseases continue to be discovered. A specific etiology was not found to be the cause of the mortalities. However, higher density in the translocation enclosures, which may induce social disruption and stress, appears to be strongly associated with the detection of clinical signs and deaths of tortoises. Other factors, such as initial body condition, may also have contributed. This retrospective study has revealed important factors that appear correlated with probability of disease, and therefore provide guidance regarding both important data to collect as part of translocation and factors to consider when planning releases. For example, more information regarding donor site, such as habitat or vegetation type and quality, were not available for most donor sites but may be important determinants of tortoise health at release, as well as for evaluating and understanding translocation success.

In Florida, the primary objective for gopher tortoise translocation is usually to avoid direct and immediate mortality due to development, with the secondary objective of reintroducing tortoises to an area where they were previously extirpated or to augment existing populations (15). While both objectives are met with translocation to Nokuse Plantation, this study has underscored the importance of both long-term monitoring health and disease following translocation and incorporating an adaptive management approach. Disease screening (or at least collection of samples for later pathogen testing if an issue subsequently arises) is an important consideration when combining tortoises from multiple source populations. In some cases, health problems that may arise after a translocation event are not a result of the translocated animals introducing diseases or pathogens, but rather that these naïve animals are exposed to local pathogens or contaminants (20, 67). Stress, such as that associated with the translocation process, is an additional factor that may lead to increased susceptibility to disease and infection in tortoise species (21, 44, 45, 49, 68). Should monitoring reveal elevated disease prevalence or mortality in a translocated or augmented population, translocation practices merit review and modification before implementation elsewhere.

Although gopher tortoises possess many characteristics that make them a difficult species for which to measure long-term post-translocation success (e.g., long-lived with long generation times) (69), continued surveillance and improvements in future translocation and release protocols can increase the likelihood of maintaining a stable population. One of the most important tasks is to ensure that the recipient site continues to be managed to maintain optimal habitat for gopher tortoises, such as through the frequent application of prescribed fire. Additionally, increased monitoring during the inactive season, particularly following prescribed burning, may also lead to early detection of tortoises in poor health, which could then receive early treatment. Managers should also ensure that density of gopher tortoises within release enclosures is maintained below a set maximum density to reduce the potential for additional chronic stress associated with social disruption, especially when combining tortoises from multiple sources or for confining to pens for multiple years. Based on 14 years of experience with large-scale gopher tortoise translocations and the findings of this mortality event, we recommend a site density of no more than 3 adult tortoises per ha, even in areas with high forage quality. This recommended maximum density is lower than another translocated population, in which 13 adults and subadults from the same source population were penned in a 1-ha enclosure for 6–9 mo. (23). However, our recommended density is higher than documented in many wild populations (0.02–3.08 tortoises/ha; all size classes) of gopher tortoises (61, 62, 70–72). The penning density we recommend may need to be reduced further pending additional research into the effects of translocation on social networks and effects of density on the health and behavior of translocated gopher tortoises.

## Data Availability Statement

The datasets generated for this study are available on request to the corresponding author.

## Ethics Statement

The animal study was reviewed and approved by University of Georgia IACUC.

## Author Contributions

SH, TN, and MA contributed to project design. RC, TN, NLS, and MA contributed to data collection. RC, SH, and NIS contributed to data analysis. RC wrote the manuscript. SH, TN, TT, NIS, NLS, and MA edited and reviewed the manuscript.

### Conflict of Interest

NS was employed by Busch Gardens. The remaining authors declare that the research was conducted in the absence of any commercial or financial relationships that could be construed as a potential conflict of interest.
