# Supplementary material for: Epidemiological Investigation of a Mortality Event in a Translocated Gopher Tortoise (Gopherus polyphemus) Population in Northwest Florida
Source: Front Vet Sci. 2020 Mar 5;7:120. doi: 10.3389/fvets.2020.00120 (PMC7067046; doi:10.3389/fvets.2020.00120)
Supplement: Supplementary file 1 [file Table_1.DOCX]

**Supplemental Table**: Akaike Information Criterion (AIC) table for top logistic regression models in each candidate set demonstrating variables for predicting detection of disease in translocated gopher tortoises (*Gopherus polyphemus*) at Nokuse Plantation, a private conservation preserve in northwest Florida. BCI = body condition index; numdscoh = number of diseased tortoises in cohort; reg = donor region; cohds = presence of disease in cohort; numcoh = number of tortoises in cohort; site = donor site; cty = donor county; dip = days in pen; seas = season found; scl = straight carapace length; daterel = date relocated; dens = initial density of release enclosure; burn1 = days since last prescribed burn; dcoh = number of different cohorts, null.m = null model (intercept only).

| Candidate Set | **Model** | **K** | **AIC** | **ΔAIC** | **Model Wt** |
| --- | --- | --- | --- | --- | --- |
| Donor Site | numdscoh | 2 | 341.428 | 0.000 | 0.725 |
|  | reg.numdscoh | 8 | 343.504 | 2.076 | 0.257 |
|  | cohds.numcoh | 3 | 348.865 | 7.438 | 0.018 |
|  | numcoh | 2 | 355.270 | 13.843 | 0.001 |
|  | reg | 7 | 359.565 | 18.137 | 0 |
|  | reg.cohds | 8 | 360.663 | 19.236 | 0 |
|  | cohds.site | 86 | 365.432 | 24.004 | 0 |
|  | null.m | 1 | 368.842 | 27.415 | 0 |
|  | cohds | 2 | 369.386 | 27.959 | 0 |
|  | cty | 37 | 376.175 | 34.748 | 0 |
|  | site | 85 | 436.342 | 94.915 | 0 |
|  |  | **K** | **AIC** | **ΔAIC** | **Model Wt** |
| Host Characteristics | bci.dip.seas | 7 | 183.405 | 0.000 | 0.459 |
|  | seas | 5 | 184.406 | 1.001 | 0.278 |
|  | bci.seas | 6 | 186.302 | 2.897 | 0.108 |
|  | scl.seas | 6 | 186.342 | 2.938 | 0.106 |
|  | scl.bci.seas | 7 | 188.198 | 4.793 | 0.042 |
|  | month | 12 | 191.786 | 8.382 | 0.007 |
|  | dip | 2 | 256.616 | 73.211 | 0 |
|  | bci.dip | 3 | 257.135 | 73.730 | 0 |
|  | null.m | 1 | 368.842 | 185.438 | 0 |
|  | scl | 2 | 370.150 | 186.745 | 0 |
|  | bci | 2 | 370.836 | 187.432 | 0 |
|  | sex | 3 | 371.324 | 187.919 | 0 |
|  | scl.bci | 3 | 372.132 | 188.728 | 0 |
|  | scl.sex | 4 | 372.180 | 188.775 | 0 |
|  | scl.sex.bci | 5 | 374.099 | 190.694 | 0 |
|  | daterel | 208 | 618.129 | 434.725 | 0 |
|  | scl.sex.daterel | 211 | 622.229 | 438.824 | 0 |
|  | **K** | **AIC** | **ΔAIC** | **Model Wt** | **K** |
| Recipient Site | dens.burn1 | 3 | 204.979 | 0.000 | 0.972 |
|  | burn1 | 2 | 212.102 | 7.123 | 0.028 |
|  | dens.dcoh | 3 | 311.977 | 106.998 | 0 |
|  | dens | 2 | 313.119 | 108.140 | 0 |
|  | dcoh | 2 | 319.520 | 114.541 | 0 |
|  | null.m | 1 | 368.842 | 163.864 | 0 |
