# Supplementary material for: Epidemiological Investigation of a Mortality Event in a Translocated Gopher Tortoise (Gopherus polyphemus) Population in Northwest Florida
Source: Front Vet Sci. 2020 Mar 5;7:120. doi: 10.3389/fvets.2020.00120 (PMC7067046; doi:10.3389/fvets.2020.00120)
Supplement: Supplementary file 2 [file Table_2.DOCX]

**Appendix A. Forage Nutrition Evaluation Procedure**

**Methods:**

Each sample was weighed and oven dried for four hours at 60 C° to measure percent moisture and percent dry matter. The following values were measured as a percentage of the dry matter in each sample: Crude Protein (CP), Soluble Protein (SP), Acid Detergent Insoluble Crude Protein (ADICP), Neutral Detergent Insoluble Crude Protein (NDICP), Acid Digested Fiber (ADF), amylase and sodium sulfite treated Neutral Detergent Fiber on organic matter (aNDFom), lignin, crude fat, ash, starch, Ethanol Soluble Carbohydrates (ESC, simple sugars), Nonfibrous Carbohydrates (NFC), Relative Feed Value (RFV), Total Digestible Nutrients (TDN), Net Energy for lactation (NEl), Net Energy for Maintenence (NEm), Net Energy for gain (NEg), Metabolizable energy (ME), Digestible Energy (DE), Calcium (Ca), Phosphorus (P), Magnesium (Mg), Potassium (K), Sodium (Na), Iron (Fe), Zinc (Zn), Copper (Cu), Manganese (Mn), Molybdenum (Mo), Sulfur (S), Chloride ion (Cl), and Dietary Cation-Anion Difference (DCAD).

Specific methods for the following analyses were taken from the Dairy One Forage Lab Analytical Procedures Manual.

ADICP: ADF residue analyzed using a Leco TruMac N Macro Determinator to determine the protein fraction bound to the acid detergent fiber.

Crude Fat: Extraction by Soxtec HT6 System using anhydrous diethyl ether. Crude fat residue determined gravimetrically after drying.

ESC: Samples shaken for 4 hours at 180 epm with 80% ethanol to extract ethanol soluble carbohydrates comprised of simple sugars. ESC determined using a Thermo Scientific Genesys 10S Vis Spectrophotometer after a colorimetric phenol-sulfuric acid reaction.

ADF: Solutions as in AOAC 973.18 – Fiber (Acid Detergent) and Lignin (H2SO4) in Animal Feed. Samples individually weighed at 0.5g into filter bags and digested for 75 minutes as a group of 24 in 2L of ADF solution in ANKOM A200 Digestion Unit. Samples are rinsed three times with boiling water for 5 minutes in filter bags followed by a 3 minute acetone soak and drying at 105ºC for 2 hours.

Lignin: Solution as in AOAC 973.18 – Fiber (Acid Detergent) and Lignin (H2SO4) in Animal Feed. ADF performed as above and residue digested as a group of 24 in 72% w/w sulfuric acid for 3 hours in ANKOM DaisyII Incubator at ambient temperature.

aNDFom: (amylase and sodium sulfite treated Neutral Detergent Fiber on an organic matter (ash free) basis) Samples individually weighed at 0.5g into filter bags and digested for 75 minutes as a group of 24 in 2L of NDF solution in ANKOM A200 Digestion Unit. Four ml of Alpha Amylase and 20g sodium sulfite are added at the start of digestion. Samples are rinsed three times with boiling water for 5 minutes. Alpha Amylase is added to the first 2 rinses. Water rinses are followed by a 3 minute acetone soak and drying at 105ºC for 2 hours. aNDF analyzed as above but with the addition of an ashing step to remove inorganic materials such as minerals, soil, and sand by burning the fibrous residue at 550C for 2 hours.

All minerals: Samples digested using CEM Microwave Accelerated Reaction System (MARS6) with MarsXpress Temperature Control using 50ml calibrated Xpress Teflon PFA vessels with Kevlar/fiberglass insulating sleeves then analyzed by ICP using a Thermo iCAP 6300 Inductively Coupled Plasma Radial Spectrometer. Samples first pre-digested at ambient temperature 10 minutes with 8ml nitric acid (HNO3) and 2ml hydrochloric acid (HCl) and then an additional 10 minutes with 1ml 30% hydrogen peroxide (H2O2). After pre-digestion complete, samples ramped to 200ºC in 15 minutes and finally held at digestion temperature of 200ºC for 15 minutes at 1600W. Vessels brought to 50-ml volume, aliquot used for analysis.

Chloride ion: 0.5g dried, ground sample or 5g wet sample extracted for 15 minutes in 50ml 0.1N HNO3, followed by potentiometric titration with AgNO3 using Brinkmann Metrohm 716 Titrino Titration Unit with silver electrode. For water samples, 25ml of 0.2N HNO3 added to 25ml of sample then analyzed.

NDICP: aNDF performed without sodium sulfite then residue analyzed using a Leco TruMac N Macro Determinator to determine the protein fraction bound to the neutral detergent fiber.

SP: Cornell Sodium Borate-Sodium Phosphate Buffer Procedure. Soy products incubated at 39°C. All other samples incubated at ambient temperature. Residue containing insoluble protein analyzed using Leco TruMac N Macro Determinator.

Starch: Samples are pre-extracted for sugar by incubation in 40ºC water bath and filtration on Whatman 41 filter paper. Residues are thermally solubilized using an autoclave, then incubated with glucoamylase enzyme to hydrolyze starch to produce dextrose (glucose). Prepared samples injected into sample chamber of YSI Analyzer where dextrose diffuses into a membrane containing glucose oxidase. The dextrose is immediately oxidized to hydrogen peroxide and D-glucono-4-lactone. The hydrogen peroxide is detected amperometrically at the platinum electrode surface. The current flow at the electrode is directly proportional to the hydrogen peroxide concentration, and hence to the dextrose concentration. Starch is determined by multiplying dextrose by 0.9.
